# Contrasting timescales of metal fluxes in porphyry copper systems from coupled physicochemical processes of magmas, rocks and fluids

**DOI:** 10.1038/s41598-025-15335-8

**Published:** 2025-08-15

**Authors:** Yulia Gruzdeva, Philipp Weis

**Affiliations:** 1 GFZ Helmholtz Centre for Geosciences, Telegrafenberg, 14473 Potsdam, Germany; 2 Institute of Geosciences, University of Potsdam, Karl-Liebknecht-Straße 24/25, 14476 Potsdam, Germany

**Keywords:** Magmatic-hydrothermal systems, Ore formation, Porphyry copper deposits, Numerical modelling, Geochemistry, Economic geology

## Abstract

Volatile degassing from hydrous magma reservoirs controls the formation of porphyry copper deposits. Geochemical studies suggest that water-rich magmas may be more prone for ore formation, with fluid-melt partitioning potentially producing particularly metal-rich fluid stages. However, the coupled physicochemical processes at the magmatic-hydrothermal transition remain elusive, because they depend on non-linear properties of magmas, fluids and rocks. For this study, we further developed a numerical model for magma convection, volatile degassing, hydraulic fracturing and fluid flow by modifying its permeability response to brecciation and introducing chemical fluid-melt partitioning. We investigate the role of intrusion depth, water content and distribution coefficients on degassing and ore formation. The results show how magmas can self-organize into distinct degassing stages with contrasting timescales of metal fluxes. Depth and water content control the amount of fluids released by an initial short-lived tube-flow outburst event, leading to brecciation and a first mineralization event in shallow porphyry-epithermal levels for high distribution coefficients. Further cooling leads to continuous fluid release at lower rates, producing a second mineralization event at deeper levels. Our results suggest that near-saturated water contents of voluminous magma reservoirs in combination with low fluid-melt distribution coefficients support the formation of large porphyry deposits.

## Introduction

Porphyry copper deposits (PCDs) are our main resources for metals like Cu, Mo and Au and form by volatile degassing from crystallizing hydrous magma reservoirs in subduction-related settings^1,2^. A major uncertainty in understanding the formation of magmatic-hydrothermal deposits lies in the complex interplay between the magma reservoir and the overlying hydrothermal system^3,4^. The formation of PCDs depends on the efficiency of metal precipitation from ascending and phase-separating magmatic fluids through permeable structures^4,5^. These pathways, in return, are dynamically influenced by magmatic fluid fluxes which are supplied by water-rich magmatic intrusions^6,7^. Hence, ore formation requires an ideal alignment of physical and chemical processes of the crystallizing magma reservoir and its feedbacks with the hydrothermal system^8–11^.

Recent studies investigated the role of intrusion depth, water content and fluid-melt distribution of chemical elements on the potential to form large PCDs^8,9^. Most interpretations include voluminous upper-crustal magma reservoirs that have been incrementally assembled as a prerequisite for ore formation^12–14^. The transfer of metals from melt to fluid is controlled by chemical competition among crystals, melt, and fluid, and is thus closely related to the crystallization process^9^. As the melt becomes saturated with volatiles, a magmatic fluid exsolves, scavenging copper through ligand interactions as $\textrm{Cu}-\textrm{Cl}$ complexes. The partitioning of copper and salt into the fluid depends on the evolving melt composition and the distribution coefficient $\textrm{D}_{\textrm{i}}=\textrm{C}_{\textrm{i}}($fluid$) / \textrm{C}_{\textrm{i}}($melt), which reflects the efficiency of this transfer. Previous works show that the bulk input chemistry is determined in the lower crust^8,9,15^, and Cu concentrations can be maximized if Cu (and S) are effectively extracted from the cooling magma to the magmatic fluid, which is partially controlled by the initial water content, chlorine concentrations and oxygen fugacity of the magma^8^. This effect of fluid-melt partitioning can be levelled out again by homogenization of volatiles from different stages during radial cooling, as shown by first-order simulations of pure conductive cooling of incrementally assembled magma reservoirs^12^. However, the physicochemical processes at the magmatic-hydrothermal transition still remain elusive due to our limited understanding of these interactions and lack of adequate tools to quantify the interplay of these non-linear processes.

Numerical models have become essential tools for studying magmatic-hydrothermal systems, linking field observations to underlying physical and chemical processes^12,14,16–18^. Recent progress in numerical modeling, particularly the integration of permeability and porosity development in cooling magmatic intrusions^19^, revealed how magmatic systems can concentrate and channel exsolved magmatic fluids into the cupola of an intrusion, occurring in the intermediate range of crystal volume fractions (CVF = 0.4-0.7^20^). Simulations that also consider a stage of magma convection at low crystallinities indicate that the magmatic-hydrothermal system can self-organize into a succession of three distinct outgassing regimes: tube-flow outburst, flushing and radial cooling^21^.

Building on these findings, this study further develops the numerical model to quantify metal transfer into magmatic fluids by adding functionality for fluid-melt partitioning with different distribution coefficients. To account for the rapid degassing events of tube-flow outburst, we further introduce a flux-controlled release mechanism, which regulates fluid release from the magma chamber defined by a maximum attainable permeability during brecciation. With this augmented model, we can investigate the effect of intrusion depths, water contents and distribution coefficients on metal fluxes and mineralization depths during the formation of PCDs.

## Model configuration

**Fig. 1:**
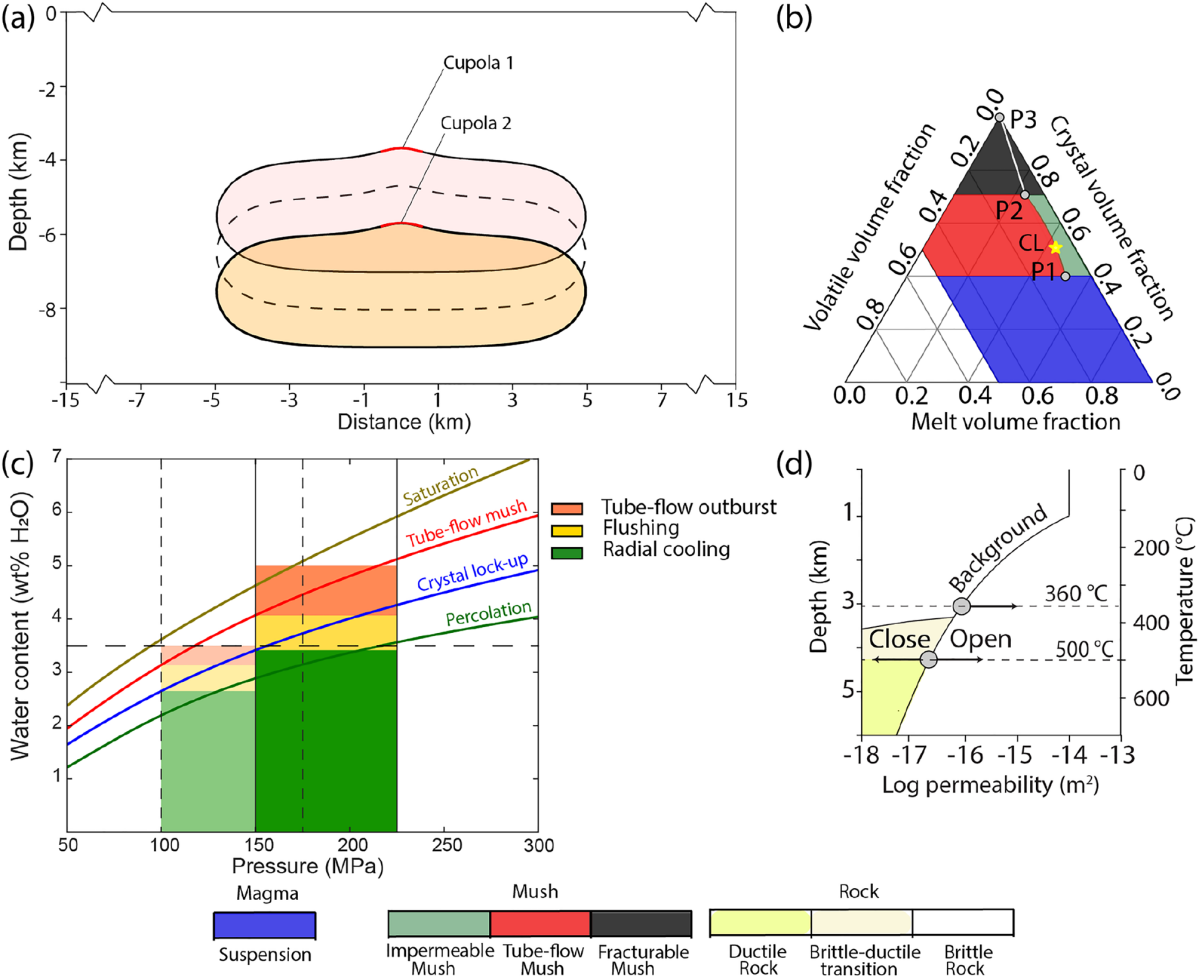
(**a**) Model geometry of a two-dimensional cross-section of the upper crust with magmatic intrusions at different depths with fluid release from a cupola region at about 4 and 6 km. (**b**) Ternary diagram showing the evolution of crystal-melt-volatile mixtures in the magma reservoir^20^, defining different stages of suspension (blue), tube-flow mush (red), impermeable mush (green) and fracturable mush (black). (**c**) Barplot showing predicted degassing stages^21^ of tube-flow outburst (orange), flushing (yellow) and radial cooling (green) for different initial water contents of the magma and depths of the cupola zones (Table 1). These stages depend on the maximum water contents at transition points in the ternary diagram (**b**) at P1 (red line; tube-flow mush), CL (blue line; crystal lock-up) and P2 (green line; percolation). (**d**) Dynamic permeability relation in the hydrothermal system of the host rock, describing permeability increase (’open’) by hydraulic fracturing and permeability decrease (’close’) at temperatures above the brittle-ductile transition^34^.

For this study, we further developed a coupled magmatic-hydrothermal model^21^ which integrates magma convection^22^, crystallization^23^, degassing^20,24^ and hydrothermal fluid flow^16^. We use a generic representation of a 2-dimensional cross-section of the upper crust with an initial magma reservoir at variable depths with an initial temperature of $970\;^{\circ }\textrm{C}$ and the volume of $\sim$50 $\textrm{km}^{3}$ (Fig. 1a). To simulate the fluid evolution in three dimensions, we use half-radial mesh volumes in reference to the vertical center axis of the model^21^, where the control volumes represent concentric rings around the center line. Figure 1b illustrates the progression of the crystal-melt-volatile mixture through successive phases^20,24^. Figure 1c shows the depth-dependence of water saturation of the initial magma (brown line) together with the maximum amounts of water contents that can be contained in the reservoir below the percolation threshold at a crystal volume fraction (CVF) $=0.4$ (red line; P 1 in Fig. 1b), at $C V F=0.5$ (blue line; crystal-lockup CL in Fig. 1b), and at CVF $=0.7$ (green line; *P*2 in Fig. 1b). A previous numerical study^21^ predicts that the values on these lines at the pressure values at the cupola region determine the amounts of fluids released by three consecutive outgassing regimes: tube-flow outburst (red field), flushing (yellow), and radial cooling (green). Figure 1d illustrates the dynamic permeability model applied to the host rock^16^. A detailed description of the model and its governing equations can be found in previous studies and datasets^16,21,22,25^.

In the methods section, we first provide an overview of the magma stages and degassing mechanisms of the model^21^. We then describe the further developments that were necessary for this study. Addition material is provided in an associated data publication ^26^.

We present a total of nine selected simulations to explore the influence of depth, water content and distribution coefficient D (Table 1). We assume that the magma reservoirs have been assembled by multiple injections at rates high enough to allow homogenization by convection. We simulate the temporal evolution of cooling and crystallizing dacitic magmas with $3.5 \mathrm {~wt} \%$ and $5 \mathrm {~wt} \% \mathrm {~H}_{2} \textrm{O}$ content following a phase of magmatic emplacement at shallower (a cupola located at 4 km depth) and deeper (at 6 km depth) levels (Fig. 1a and c). We first use a constant bulk composition of the magmatic volatiles with 500 ppm Cu and $10 \mathrm {~wt} \% \mathrm {~NaCl}$. We then use the same initial bulk compositions, but apply D values of 5 and 50, respectively, during the evolution.

**Table 1:** Summary of input parameters used for the numerical simulations.

Case	Water content (wt% $\textrm{H}_{2} \textrm{O}$)	Cupola depth (km)	Distribution coefficient D
1a	3.5	4	-
1b	3.5	4	5
1c	3.5	4	50
2a	3.5	6	-
2b	3.5	6	5
2c	3.5	6	50
3a	5.0	6	-
3b	5.0	6	5
3c	5.0	6	50

## Results

### Early degassing by tube-flow outburst and flushing

**Fig. 2:**
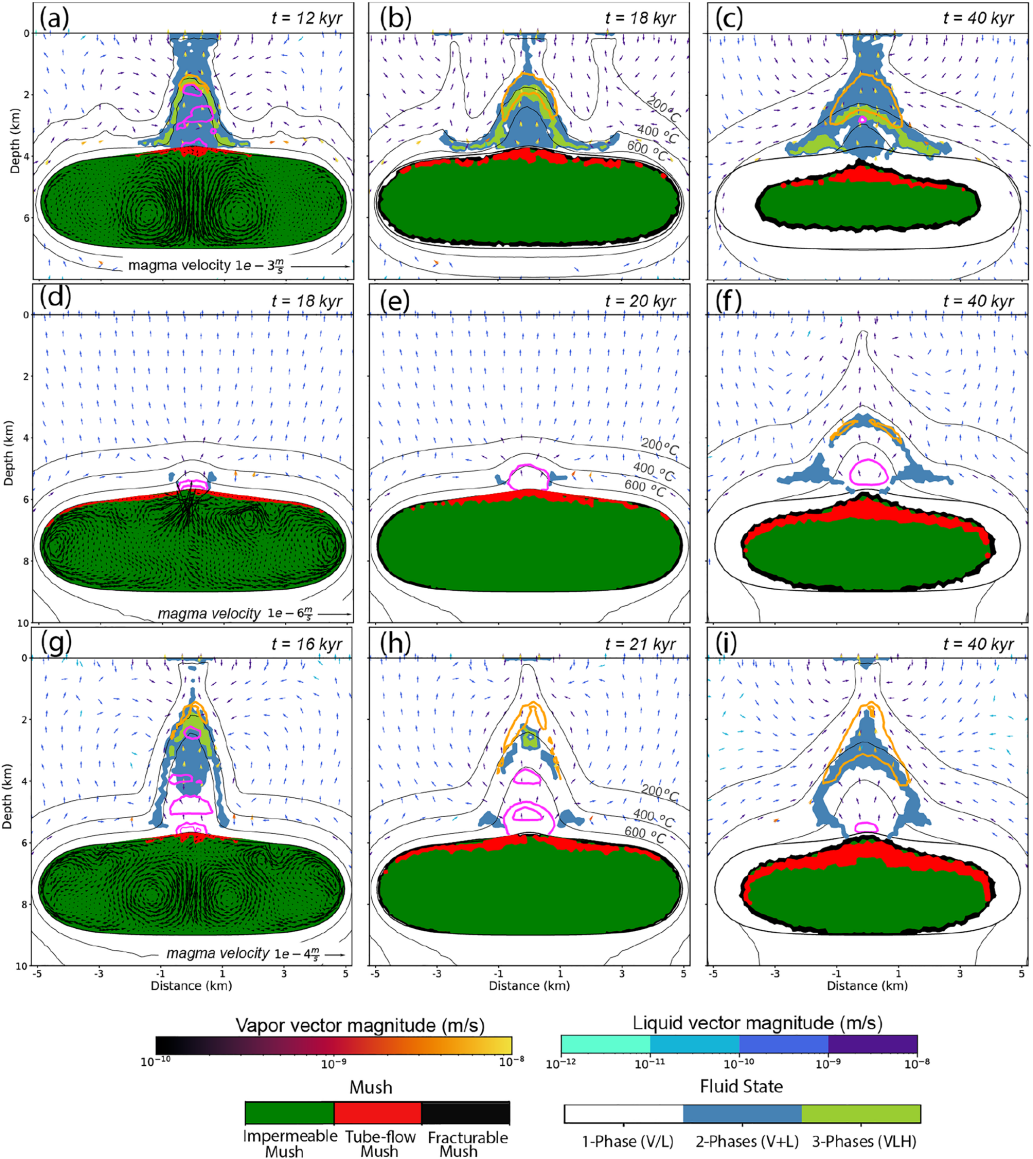
Temporal evolution of the magmatic-hydrothermal system for cases 1a (**a**–**c**), 2a (**d**–**f**), and 3a (**g**–**i**), showing the effect of magma outgassing stages via tube-flow and fracturable mush regimes on the hydrothermal system for a shallow intrusion. Colours in the partially molten magma reservoir show the respective stage in the ternary diagramm (Fig. 1b). In the mush regions, the model assumes that the magma is still mobile before crystal lock-up and can convect at CVFs between 0.4 and 0.5. Colours in the host rock show the respective fluid state (1-, 2- or 3-phase; L = liquid, V = vapor, H = halite). Orange contour lines show copper enrichment factors of 100; pink contour lines show values of additional porosity of 2% created by hydraulic fracturing.

In the initial stage following the emplacement of a magmatic intrusion at 4 km depth with a near-saturated water content of $3.5 \mathrm {~wt} \% \mathrm {~NaCl}$ (case 1a; Table 1 and Fig. 1), the temperature contrast between the cooler host rock and the partially molten magma drives convection within the reservoir (Fig. 2a). This process gradually cools the intrusion and promotes mixing within its interior. During the suspension stage at CVF $<0.4$, magma convection helps to retain exsolved magmatic fluids within the reservoir because the velocity of magma flow exceeds buoyancy-driven bubble ascent. The homogenization effect results in a rapid transition from a suspension state (blue area in Fig. 1b) to a mush state (green and red areas in Figs. 1b and 2a) at a temperature of $760 \;^{\circ }\textrm{C}$. Due to the pressure-dependence of water saturation in the melt (Fig. 1c), magmatic volatiles accumulation is highest at the zone of lowest pressure at the cupola. Where the volatile volume fraction (VVF) exceeds the percolation threshold (P1-P2) between impermeable (green area) and a tube-flow mush (red area), volatiles migrate upwards to the point of lowest pressure.

**Fig. 3:**
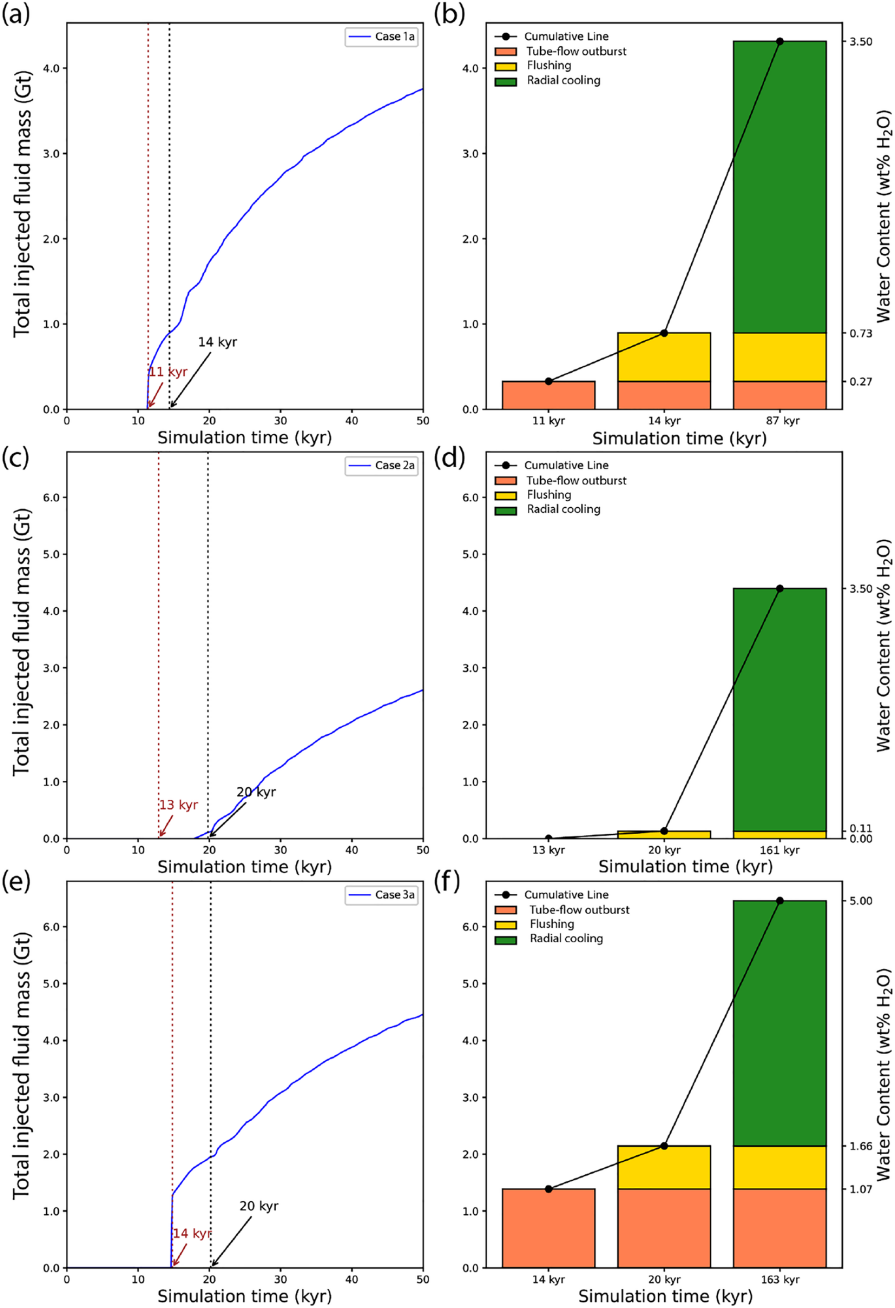
Cumulative plot of injected magmatic fluids of cases 1a (**a**), 2a (**c**) and 3a (**e**) with corresponding bar charts showing the amount of magmatic fluids released from the intrusion during different outgassing stages (**b**, **d**, **f**) as indicated in Fig. 1c.

**Fig. 4:**
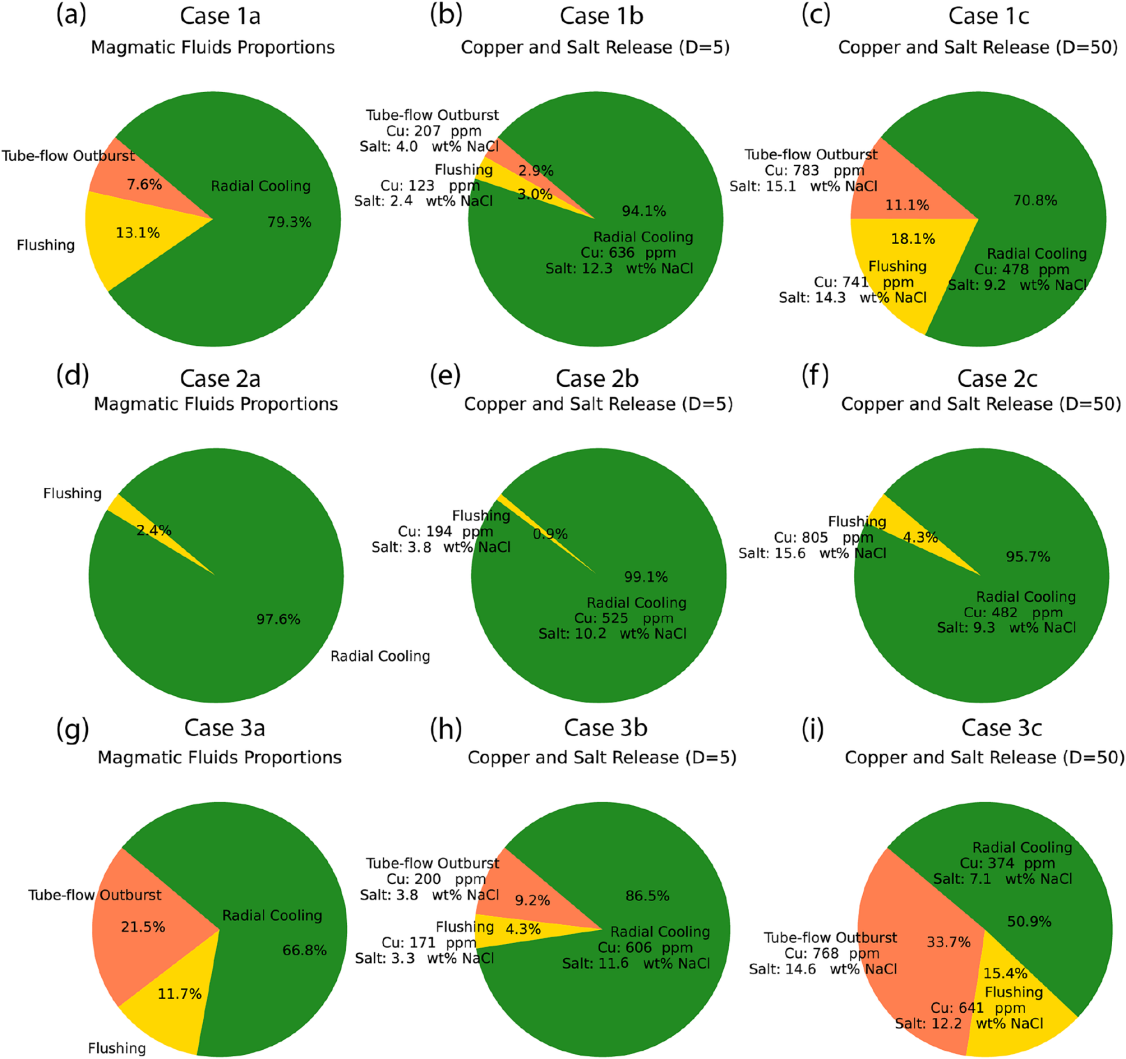
Magmatic fluid degassing at different stages with magmatic fluid proportions with constant fluid compositions of 500 ppm Cu and 10 wt% NaCl (**a**, **d**, **g**) and distribution of copper and salt proportions with high (D = 50; **b**, **e**, **h**) and low (D = 5; **c**, **f**, **i**) distribution coefficients, showing average fluid compositions for the different outgassing phases.

Fluid release starts with an outburst of early magmatic fluids from the cupola at 11 kyrs (Fig. 3a). The massive outflow from tube-flow mush constitutes $7.6 \%$ of the total volume of magmatic fluids within $\sim 100$ years (orange field in Figs. 3b and 4a). At CVFs between 0.4 and 0.5, the magma mush is still assumed to be mobile, albeit at lower velocities due to a steady increase of magma viscosity with cooling and crystallization. During this stage, VVFs are below the percolation threshold (green area) in most of the reservoir, except for the uppermost region underneath the cupola, where tube-flow mush (red area) can develop. The system now establishes a second degassing mechanism by flushing exsolved volatiles with magma convection to the cupola zone (Fig. 2a), with an additional $13.1 \%$ of the total water content being released within $\sim 3000$ yrs (yellow field in Figs. 3b and 4a). The simulated amounts of fluid release by tube-flow outburst and flushing roughly agree with the predicted values from the theoretical approximation (Fig. 1c).

Hydraulic fracturing as a response to the rapid release of magmatic fluids from tube-flow mush creates permeable pathways in the host rock overlying the cupola region. Figure 2a shows the moment of the early magmatic fluids outburst event, which generates a P-T anomaly within the central area of the hydrothermal system above the cupola, resulting in rapid heating between the magmatic intrusion and the surface. Magmatic volatiles are injected as a two-phase vapor-brine mixture (light blue area) and also vent at two-phase conditions at the surface that resemble volcanic fumaroles. Depressurization during ascent leads to precipitation of a solid halite phase (light green area) and further ascent of a vapor-dominated magmatic fluid mixture. The near-explosive character of magmatic fluid release related to the transition from suspension to tube-flow mush leads to the development of additional porosity (pink contours in Fig. 2a–i). The flow of magmatic vapor and brine through this two-phase region maintains temperatures of $>500\;^{\circ }\textrm{C}$ in the upflow zone. The formation of a copper shell, indicated by an orange contour line for a copper enrichment factor (CEF) of 100 (Fig. 2a), is associated with a temperature decrease of the magmatic fluids to $350-450 \;^{\circ }\textrm{C}$. Early copper precipitation occurs from coexisting fluid, vapor and halite phases at shallow depths of $<2 \mathrm {~km}$.

### The effect of intrusion depth and water content on early degassing

Changing the intrusion depth from 4 to 6 km in case 2a affects the outgassing behavior and fluid evolution. The transition from suspension to mush occurs slightly later at 13 kyrs (Fig. 3c), because the temperature gradient to the surrounding rock is slightly lower at greater depth. However, because water saturation of the melt is depth-dependent (Fig. 1c), VVFs are too low for a tube-flow outburst event and initial outgassing sets in later by flushing at $\sim 18$ kyrs (yellow field in Fig. 3d). As predicted (Fig. 1c), fluid release by flushing only constitutes 2.4 % of the total volume of all magmatic fluids (Fig. 4d). In contrast to case 1a, magmatic volatiles are injected as a single-phase fluid with initially only limited phase separation at the sides of the magmatic plume (Fig. 2d). The lower injection rate limits the ability of magmatic fluids to fracture the host rock, reducing the potential for the formation of effective fluid pathways and copper vein formation.

Increasing the water content from $3.5 \mathrm {~wt} \%$ $\textrm{H}_{2} \textrm{O}$ to a near-saturated water content at 6 km depth of $5 \mathrm {~wt} \%$ $\textrm{H}_{2} \textrm{O}$ in case 3a initiates a first fluid release after 14 kyrs from tube-flow mush similar to case 1a in an outburst event at the suspension-mush transition (Figs. 2g and 3e). The amount of fluids released by tube-flow outburst and flushing, which comprises $21.5 \%$ and $11.7 \%$ of total magmatic fluids, respectively (Fig. 4g), is in agreement with the predicted approximations (Fig. 1c). The tube-flow outburst event exhibits vapor venting at the surface (fumaroles; Fig. 2g). Even though the intrusion of case 3a ($5 \mathrm {~wt} \%$ $\textrm{H}_{2} \textrm{O}$, 6 km) is located 2 km deeper than the one from case 1a ($3.5 \mathrm {~wt} \%$ $\textrm{H}_{2} \textrm{O}$, 4 km), the initial zone of copper precipitation is located at $<2 \mathrm {~km}$ depth in both cases (Fig. 2a and c). The geometry of the copper shell follows the $400\;^{\circ }\textrm{C}$ isotherm and is relatively narrow. Its shape is controlled by the balance between magmatic fluid expulsion and hydrothermal convection patterns. Once the magmatic fluid plume has been established, the release rate of magmatic volatiles is controlled by the maximum permeability during hydraulic fracturing and the cooling rate by meteoric fluid convection at shallow crustal levels, as well as phase relations within the multi-phase fluids.

### Continuous degassing during radial cooling

After 14 kyrs, the magmatic system of case 1a ($3.5 \mathrm {~wt} \%$ $\textrm{H}_{2} \textrm{O}$, 4 km) reaches a CVF of $50 \%$ within the entire reservoir (Figs. 2b and 3a). Crystal lock-up prohibits further convective motion and the system transitions to a stage of radial cooling (Fig. 2b,c). During this phase of conductive cooling within the reservoir, the crystallization front advances inward, and additional outgassing occurs from rings of fracturable mush (black area) and to a lesser extent from tube-flow mush (red area). During the flushing phase of exsolved volatiles to the cupola region where the mush was still mobile, most of the volumes of the reservoir have remained in an impermeable mush state. After transition to radial cooling, the center part only cools slowly and hence remains in this impermeable mush state until it gets effected by the inward cooling progression.

With decreasing volatile release rates, meteoric water infiltrates the system and causes the magmatic plume to retreat, as illustrated by the downward movement of isotherms with time (Fig. 2b,c). Gradual cooling of the hydrothermal system also extends the copper precipitation zone downwards, leading to the extension of a precipitation zone that overprints the early mineralized zone by late fluids expelled from the fracturable mush. In case 1a, the majority of the fluids (79.3%) are injected during this later stage of radial cooling, which lasts <50 kyrs (Fig. 4a). Compared to the outburst from tube-flow mush, fluid release from fracturable mush can be produced at any depth of the reservoir and occurs at a lower rate due to the lack of preconcentration mechanism (or incubation period) during the convection-suspension period.

In case 2a ($3.5 \mathrm {~wt} \%$ $\textrm{H}_{2} \textrm{O}$, 6 km), 97.6% of the fluids are released during radial cooling (Fig. 4d). The brittle-ductile transition (BDT) is only slightly moved to shallower levels due to release from fracturable mush after 20 kyrs (Fig. 2e), eventually forming a narrow two-phase ($V+L$) zone (Fig. 2f). In this case, the system forms a thin ore shell (CEF $=100$ ) at a depth of $\sim 4 \mathrm {~km}$, where the temperature ranges from 350 to $450 \;^{\circ }\textrm{C}$.

In case 3a ($5 \mathrm {~wt} \%$ $\textrm{H}_{2} \textrm{O}$, 6 km), the total amount of fluids released by radial cooling is 4.3 Gt (Fig. 3e,f), which is the same as in case 2a ($3.5 \mathrm {~wt} \%$ $\textrm{H}_{2} \textrm{O}$, 4 km) but represents only $66.8 \%$ of the total amount of water content (Fig. 4g). With a reduced fluid release rate during the flushing and radial cooling stages, the ore shell gradually retreats to a depth of ca. 4 km , similar to case 2a, resulting in a vertically extensive zone of mineralization (Fig. 2i).

### Contrasting copper and salt fluxes

**Fig. 5:**
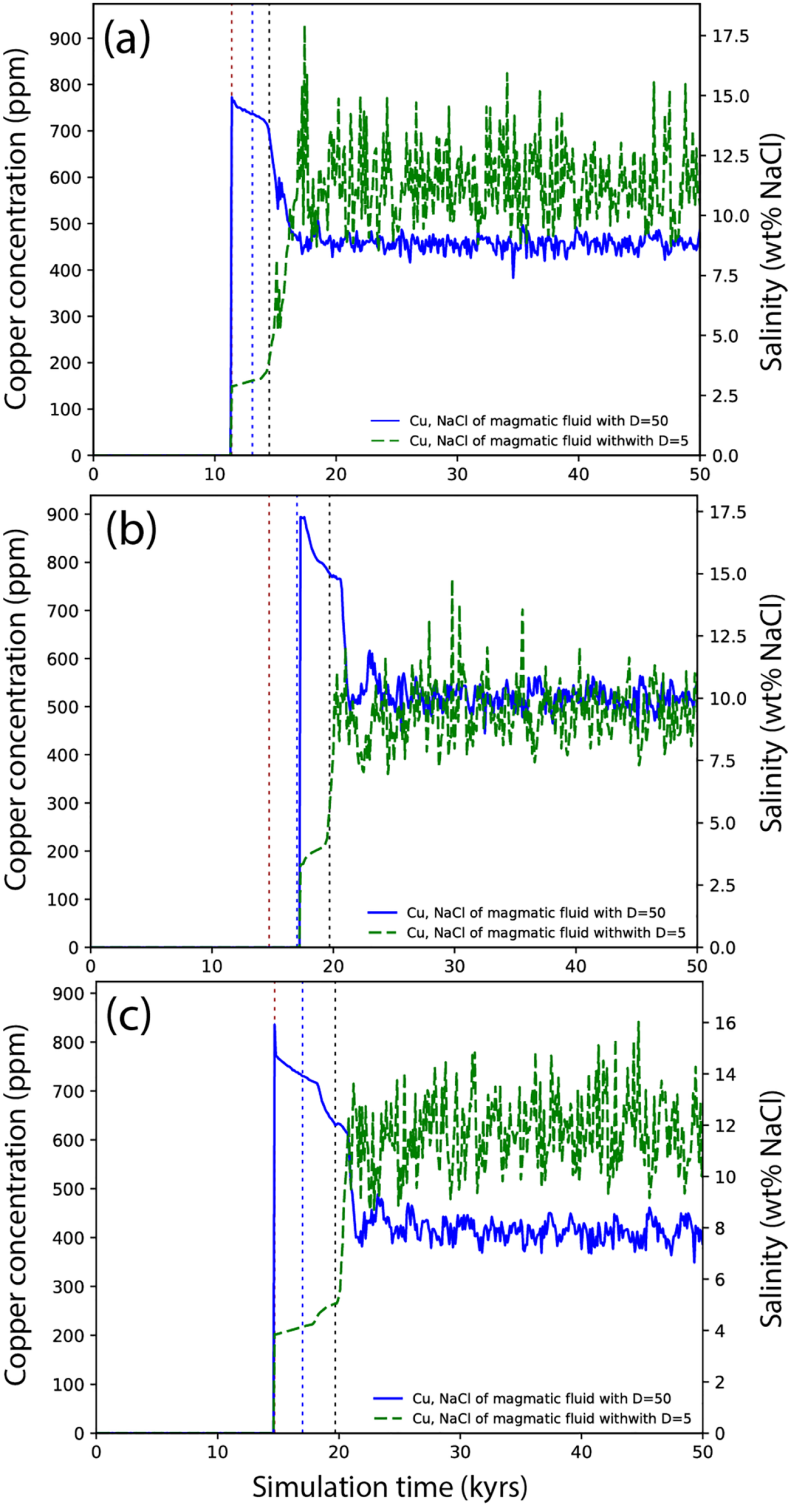
Temporal evolution of copper concentrations and salinities of the released magmatic fluids using different distribution coefficients of D = 50 (blue lines) and D = 5 (green lines) for cases 1b-c (**a**), 2b-c (**b**), and 3b-c (**c**). Note that for the given axes the respective lines for copper (left) and salt (right) overlap, because we use the same D coefficients. Vertical dashed lines show the timing of the magma transition to impermeable or tube-flow mush (brown), crystal lock-up at CVF = 0.5 (blue) and transition to fracturable mush (black).

The development of distinct physical degassing stages can affect the chemical composition of early- and late-stage fluids and thereby the formation of the ore shells. Figure 5 shows the temporal evolution of salinities and copper contents of the release fluids for distribution coefficients of $D=5$ (green lines) and $D=50$ (blue lines). All curves show the highest concentrations for the earliest fluids when copper and salt are ”enthusiastically fluid-loving” $(D=50)$ and the lowest concentrations when they are ”reluctantly fluid loving” ($D=5$).

Following tube-flow outburst, the average copper concentrations and fluid salinities change rapidly with either increasing or decreasing values, before transitioning to a more gradual evolution during the flushing stage. After crystal lockup, the fluids experience a second abrupt change in composition, followed by fluctuating values that on average remain relatively constant. This phase is related to radial cooling and the fluctuations are attributed to remaining mesh effects despite the more gradual fluid release from fracturable mush (line P2-P3 in Fig. 1c). The fluctuations in fluid production during radial cooling arise from the mesh sensitivity of fluid release at the magma chamber rims. Fluid production becomes localized due to fast cooling at the rims of the chamber in comparison to slow cooling in the core of the magma body. The degassing responds non-linearly to cooling with the highest production rates corresponding to the final cooling stages of individual nodes.

The average chemical composition of fluids released through different degassing stages depend on both the mass of fluids associated with a particular degassing regime and the distribution coefficient. For example, in case 1a ($3.5 \mathrm {~wt} \%$ $\textrm{H}_{2} \textrm{O}$, 4 km), tube-flow outburst accounts for $7.6 \%$ of the total fluid (Fig. 4a). The amount of copper and salt in these fluids varies from $2.9 \%$ for $\textrm{D}=5$ (case 1b, Fig. 4b) with about 200 ppm Cu and $4 \mathrm {~wt} \% \mathrm {~NaCl}$, to $11.1 \%$ for $\textrm{D}=50$ (case 1c, Fig. 4c) with about 800 ppm Cu and $15 \mathrm {~wt} \% \mathrm {~NaCl}$. For the deeper system with higher water contents of case 3a ($5 \mathrm {~wt} \%$ $\textrm{H}_{2} \textrm{O}$, 6 km), tube-flow outburst becomes even more significant, contributing $21.5 \%$ of the total fluids (Fig. 4g). In this case, the amount of copper and salt released by tube-flow outburst varies between $9.2 \%$ (case 3b, D = 5, Fig. 4h) and 33.7% (case 3c, D = 50, Fig. 4i). The following flushing stage follows the same trend, with average copper concentrations and salinities increasing or decreasing depending on the D-value.

When reaching the radial cooling stage, the average copper concentration and salinity of the fluids is either increased or decreased depending on the amount of previous degassing. During radial cooling, fluids from a wide range of temperatures from crystal lock-up to solidus are mixed and homogenized during volatile release, resulting in near-constant compositions. For cases $2 a-c$ ($3.5 \mathrm {~wt} \%$ $\textrm{H}_{2} \textrm{O}$, 6 km), fluid release is dominated by radial cooling ($99.1 \%$ and $95.7 \%$ respectively) and the early fluid stage only has a small effect on the overall mass balance, with compositions of $480-530 \mathrm {~ppm} \mathrm {~Cu}$ and $9.3-10.2 \mathrm {~wt} \% \mathrm {~NaCl}$ (Fig. 4e-f). In contrast, the difference is most pronounced for magmas with a near-saturated water content and large amounts of tube-flow outburst, producing fluids of $12 \mathrm {~wt} \% \mathrm {~NaCl}$ and 600 ppm in case 3b ($5 \mathrm {~wt} \%$ $\textrm{H}_{2} \textrm{O}$, 6 km, D = 5) or $7.1 \mathrm {~wt} \% \mathrm {~NaCl}$ and 400 ppm Cu in case 3c ($5 \mathrm {~wt} \%$ $\textrm{H}_{2} \textrm{O}$, 6 km, D = 50; Fig. 4h-i).

**Fig. 6:**
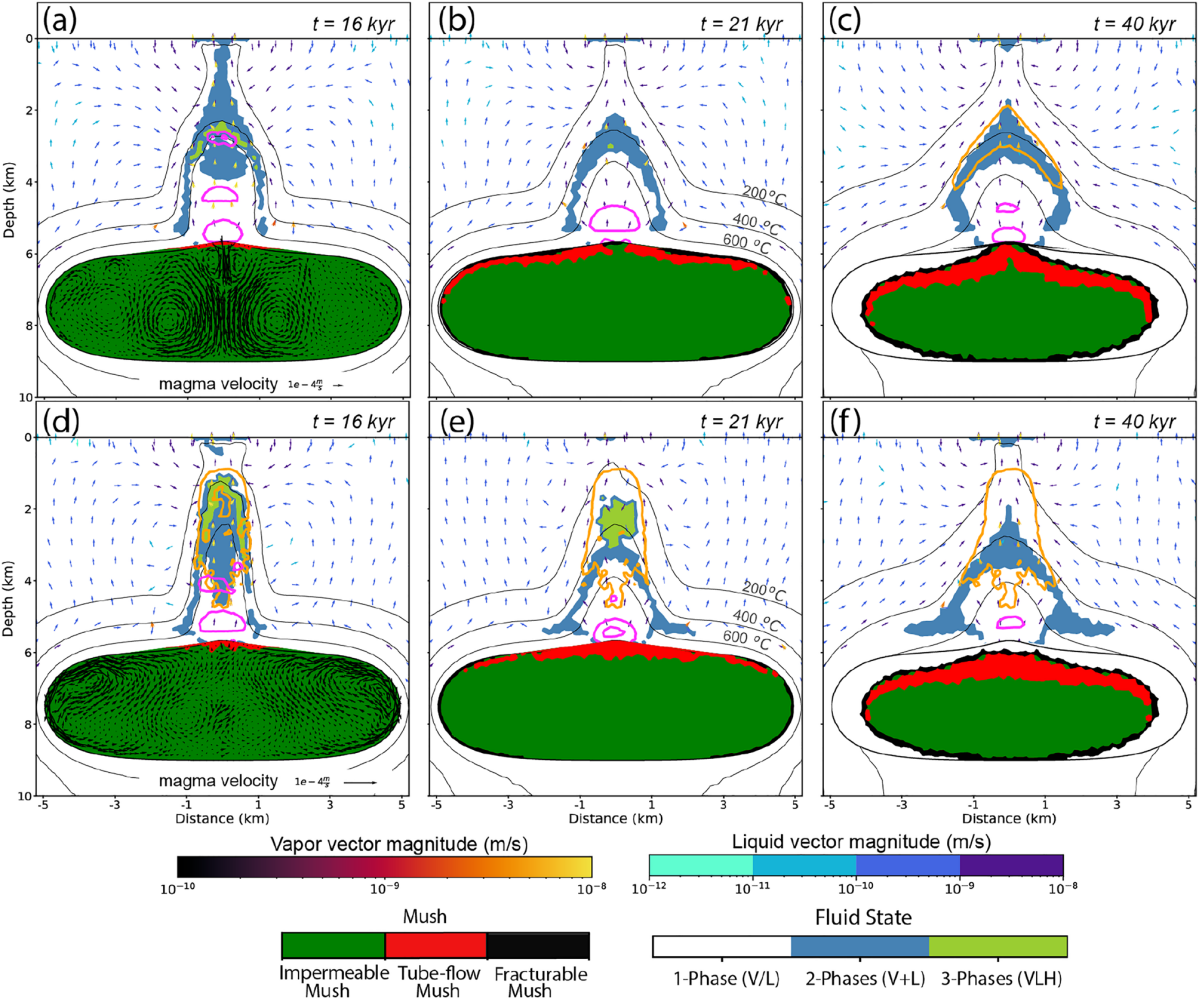
Differences in phase separation behavior within the magmatic-hydrothermal system due to different salinities of the released magmatic fluids for case 3b with D = 5 (**a**–**c**) and case 3c with D = 50 (**d**–**f**) after 16, 21, 40 kyr. Legend as in Fig. 2.

The variations in fluid salinity in cases 3b and 3c also affect the physical fluid flow, because they control the fluid properties and the behavior during phase separation upon ascent (Fig. 6a–f). Even though the fluid salinity is decreased during tube-flow outburst in case 3b (D = 5), the pressure drop marked by the halite-saturated zone (Fig. 6a) is located at $\sim 3 \mathrm {~km}$, which is deeper compared to case 3a (Fig. 2a), and phase separation of the magmatic fluid starts at a shallower depth. As the overall release rate is unchanged, the deeper location of halite saturation can be explained by a more effective cooling mechanism of meteoric convection due to a reduced clogging effect of temporarily precipitated halite. In contrast, the higher salinities during tube-flow outburst in case 3c (D = 50, Fig. 6d) lead to a narrower magmatic plume reaching to shallower depths of $<2 \mathrm {~km}$ and meteoric convection can only gradually reduce the large amounts of precipitated halite (Fig. 6e,f).

### Ore formation

**Fig. 7:**
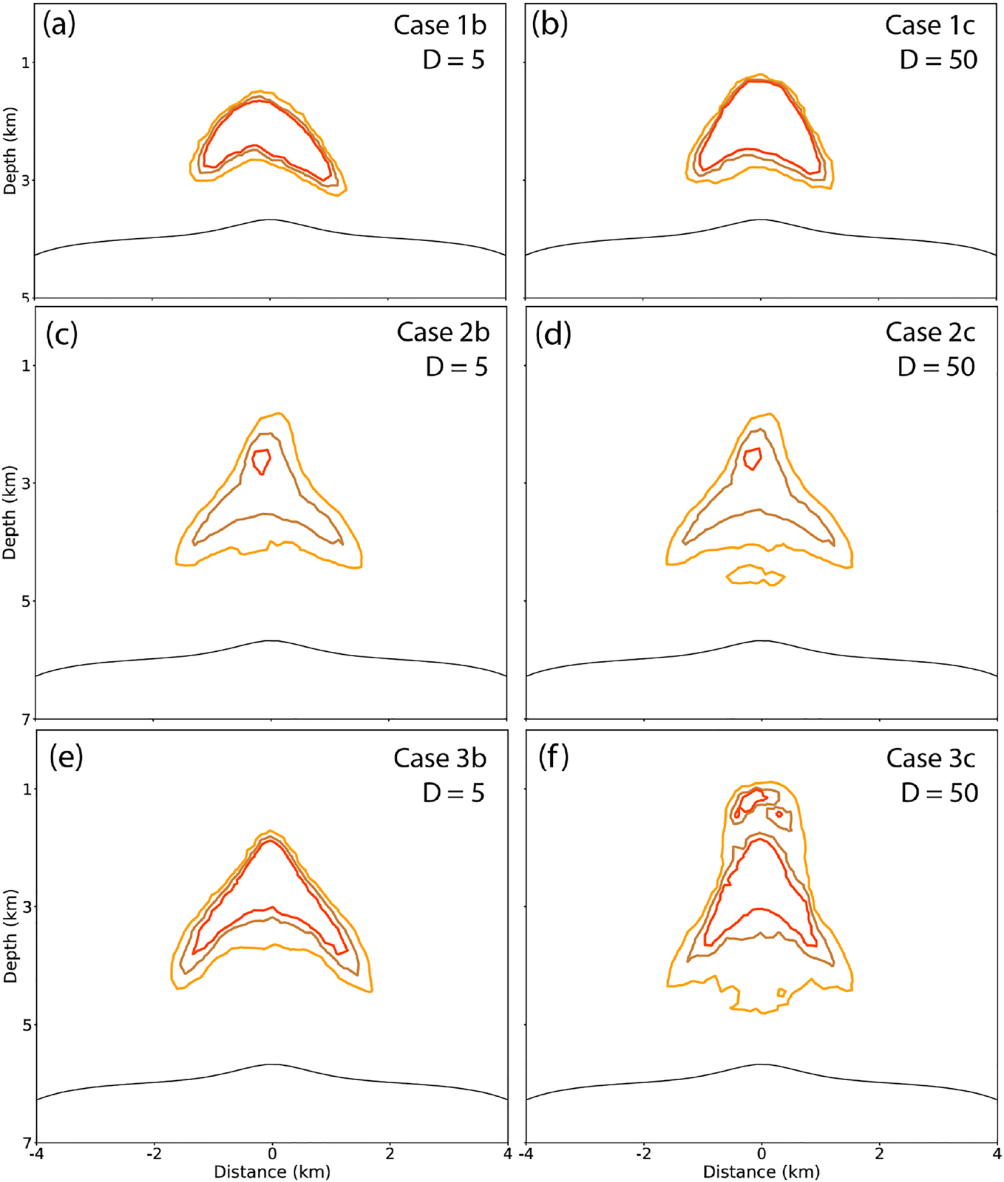
Simulated final ore shells of porphyry copper deposits, quantifying the effect of different distribution coefficients D = 5 (**a**,**c**,**e**) and D = 50 (**b**,**d**,**f**) on ore formation for cases 1b-c (**a**,**b**), 2b-c (**c**,**d**) and 3b-c (**e**,**f**) with copper enrichment factors of 100 (orange), 200 (brown), and 300 (red).

The results of the final shape and location of the ore shells show that the applied distribution coefficients only have a minimal effect for the simulations with $3.5 \mathrm {~wt} \% \mathrm {~NaCl}$ (Fig. 7a-d). In both cases, the fraction of fluids explosively expelled is significantly smaller than the fraction expelled during radial cooling.

In contrast, cases 3b and 3c ($5 \mathrm {~wt} \%$ $\textrm{H}_{2} \textrm{O}$, 6 km) with a greater amount of fluid release by tube-flow outburst, produce different ore shells for different *D* values. For ”reluctantly fluid-loving” ($\textrm{D}=5$) systems, the ore shells are nested around the central upflow zone (Fig. 7e). For ”enthusiastically fluid-loving” systems with the high D values of 50 (Fig. 7f), the ore shell is more vertically elongated, forming two zones of elevated copper enrichment (red contour corresponds to CEF = 300). The upper shell located at depth of $\sim 1 \mathrm {~km}$ is formed due to explosive outgassing (Fig. 6d) and due to elevated Cu concentrations of $\sim 840$ ppm (Fig. 4i) in the expelled early fluids (Fig. 7d), which comprises $\sim 1/3$ of the total Cu budget. Pervasive fracturing of the host rock during tube-flow outburst helped to bring Cu-rich magmatic fluids to shallow levels at the transition from shallow-porphyry to high-sulfidation epithermal conditions, forming a first low-volume but high-grade ore shell. After the retreat of the magmatic plume, the second high-grade ore shell starts to form at a similar location as for low D values. Due to the reduced copper concentration in the late-stage fluids, the copper grade for the second ore shell is reduced compared to case 3b (Fig. 7e).

## Discussion

The simulations show that intrusion depth and water content of the magma are critical parameters for the degassing evolution of magmatic intrusions that can initially be homogenized by magma convection. Water-rich magmas that reach water saturation in low-crystallinity suspension phase tend to accumulate volatiles in the cupola zones of an intrusion, which may release enough of mechanical energy to result in increased overpressure in a tube-flow outburst event as in cases 1a-c ($3.5 \mathrm {~wt} \%$ $\textrm{H}_{2} \textrm{O}$, 4 km) and 3a-c ($5 \mathrm {~wt} \%$ $\textrm{H}_{2} \textrm{O}$, 6 km). In extreme cases, fluid saturation may also trigger volcanic eruptions^27^, which cannot be captured by our current model. The simulations confirm that the amount of fluids present in different consecutive outgassing regimes can be estimated with a simple approximation diagram, which also extends to deeper intrusion levels and higher water contents (compare bar plots in Fig. 2 with Fig. 1c; see detailed description in ref^21^). Previous simulations with higher water contents of up to $7\mathrm {~wt} \%$ $\textrm{H}_{2} \textrm{O}$ show that tube-flow outburst becomes even more intense in accordance with the generic diagram and would further enhance the differences between chemical compositions of early and late degassing. Different sizes of the magma reservoir may influence fluid release rates and cooling timescales, but the relative proportions of early and late degassing will be determined by the cupola depths. A key factor for the depth of ore formation is the dynamic permeability evolution of the host rock. The model suggests that tube-flow outburst, associated with high-water content magmas, could possibly lead to breccia formations which facilitate upward transport of magmatic fluids within the host rock and can cause mineralization at shallow depth levels.

The timescales and fluid fluxes of these three different regimes differ by orders of magnitude: 1) Outgassing by tube-flow outburst is modelled to last on the order of 100 years and might be controlled by a the permeability that can be dynamically generated during hydraulic fracturing (set to $k_{\max }=10^{-13}$ $\textrm{m}^{2}$ in this study); 2) The following flushing regime at intermediate crystallinities lasts on the order of 1000s years; 3) The remaining water content is undergoing outgassing through fracturable mush at stages of radial cooling, which lasts on the order of 10,000s years. The variability of the early degassing mode impacts the depth to which the initial magmatic plume can reach by physical fracturing, hence the formation of PCD in shallow-porphyry conditions is expected to occur during tube-flow outburst of magmatic fluids. This location is independent from the intrusion depth and is rather controlled by the balance of fluid release rate and the cooling efficiency of meteoric water convection (Fig. 2a–f).

The contrasting timescales of metal fluxes develop by self-organization of the magmatic-hydrothermal system due to the interaction on non-linear properties of magmas, rocks and fluids. The modelled degassing times suggest that seemingly contrasting evidence of events lasting on the order of 100 yrs^28^ and 10s kyrs^29^ can be explained by physicochemical interactions. The modelled system also self-organizes into hydrothermal brecciation events without external triggers such as earthquakes, injection of new magma batches or volcano sector collapses^30^. However, such events may nevertheless occur and may influence the system, which could be addressed by future simulation studies. Also, the simplifying setup of the temporal evolution of a single voluminous magma reservoir cannot address the evolution of large magmatic complexes hosting giant porphyries, where studies indicate much longer timescales of several Mio years^31^.

The difference in copper content released under different outgassing regimes differs by less than an order of magnitude across the tested D-values (5, 50) due to homogenizing effects during fluid release. In particular, radial cooling from a non-convecting crystal-rich magma is predicted to result in mixing of fluids generated at different temperature and crystallinity stages. As a result, the copper content remains similar throughout the crystallization process regardless of the D-value in water-poor systems without an initial tube-flow outburst and flushing stage, similar to results from pure conductive models^12^.

In contrast, magmas with near-saturation water contents with explosive outgassing either have low or high concentrations in the early fluids, depending on the D value^9^. Systems with high D values and water contents produce two outgassing events, both with considerable metal contents, leading to two mineralization events at different depths. The ore shells show internal zonation that reflect large vertical migration, potentially explaining the gradual decline of a magmatic plume documented by lower-temperature vein and alteration assemblages overprinting earlier higher-temperature ones^32^. In systems with low D-values only a moderate amount of copper is initially dispersed during tube-flow outburst and flushing and later magmatic degassing from radial cooling concentrates the remaining copper. The following continuous degassing forms a confined ore shell with elevated copper concentration due to the longer time of outgassing at radial cooling stage.

The coupled model still assumes that all magmatic volatiles are eventually released by fracturing of the high-crystallinity mush (line P2-P3 in Fig. 1b), while in real-world systems some volatiles may stay trapped in the pore space of impermeable mush^33^. With constant low D-values, these fluids would be particularly metal rich. However, studies using experimentally constrained D-values show that they can vary during crystallization, with some configurations showing peaks in metal contents at intermediate stages^9^, which may thus be the most favorable conditions for ore formation. Future modelling studies using more realistic fluid-melt partitioning and additionally resolving the porosity and permeability evolution within the reservoir^19^ will allow for a more detailed picture of volatile release and ore formation.

## Conclusions

The presented study quantitatively links a physical degassing model^21^ based on experimental research^20,24^ to geochemical implications of chemical partitioning of copper and salt from melt to magmatic fluids^9^ for different intrusion depths and water contents.

Integrating both magmatic and hydrothermal processes into a coupled numerical model has important implications for understanding the formation mechanisms of porphyry deposits:

- The physical impact and timescales of expelled fluids on hydrothermal part is directly related to the depth and water content of the magmatic intrusion. A short-lived tube-flow outburst event only develops in water-rich systems, providing preferential pathways for later expelled fluids at the later radial cooling stage. Our simulations suggest that magmas with near-saturated initial water contents can generate three distinct fluid release mechanisms.
- Our simulations indicate that fluid release from water-rich magmas with high fluid-melt partitioning can be extended to shallow-porphyry environments with subsequent retreat to greater depths. In contrast, water-rich magmas with low D-values have the highest potential to generate high-grade confined ore shells typical for porphyry copper systems.

## Methods

### Magma stages and degassing mechanisms

For the magma part of the model, we adopted the description of different magma stages during cooling from studies on pore-scale processes^20,23,24^ based on crystal volume fractions (CVFs) and volatile volume fractions (VVFs):

- Suspension (CVF < 0.4): in this state of low-crystallinity mobile magma, volatiles can exsolve when the melt reaches water saturation, but the volatile bubbles remain in suspension due to convection, which exceeds bubble ascent velocities.
- Intermediate-crystallinity mush (0.4 < CVF < 0.7): in this state, the fluid behavior depends on whether the VVF exceeds a percolation threshold (PT) for the formation of tube-flow channels:
  - Impermeable mush (VVF < PT): magma is in a mush state but the volatiles cannot form tube-flow channels required for fluid migration. If the magma is still mobile at CVF < 0.5, the trapped volatile bubbles are still transported by magma convection as in the suspension state.
  - Tube-flow mush (VVF < PT): in this state, tube-flow channels can develop and allow for rapid degassing. In the model, we assume that the amount of volatiles above the PT can flow through the permeable tube-flow mush to the cupola region and be released to the host rock.
- Fracturable mush (CVF < 0.7): in this state of highly crystalline magma, volatile release occurs only via capillary fracturing under overpressure. In the model, we assume that all volatiles can eventually be released to the host rock, by gradually lowering the PT to a value of 0.0 at the solidus (CVF = 1.0).

In transient simulations, several of these magma stages can occur at the same time in different parts of the reservoir. Magma evolution and volatile degassing during cooling and crystallization is strongly influenced by convection, which we assume to cease at crystal-dominated conditions. In the degassing part of the model, we therefore first distinguish between different degassing stages during homogeneous and radial cooling.

- Homogeneous cooling occurs before crystal lockup (CVF < 0.5) due to advective heat redistribution by magma convection. In combination with the constraint that outgassing at intermediate crystallinities is limited to tube-flow mush, we further subdivide this stage by different fluid outgassing mechanisms:
  - Tube-flow outburst is a short-lived, high-flux degassing event, occurring in water-rich magmas during the transition from suspension to tube-flow mush at the cupola region when VVF exceeds the percolation threshold at CVF = 0.4. At the same time, the magma reservoir at greater depth can be in a state of impermeable mush due to the pressure-dependence of water saturation (Fig. 1c). All the exsolved fluids above the percolation threshold (P1 in Fig. 1b) from the entire magma reservoir are now assumed to flow towards the cupola and are released to the host rock.
  - Flushing follows the tube-flow outburst event and occurs before lock-up in the state of intermediate crystallinity by ongoing magma convection. The model assumes that the mush is still mobile at 0.4 < CVF < 0.5. As a consequence, the cupola region is kept in a state of tube-flow mush, where fluids can form tube-flow channels and physically separate from the mush. Most of the magma volume remains in a state of impermeable mush but exsolved volatiles can be transported (“flushed”) with the convecting magma to the cupola region where the volatiles in excess of the percolation threshold are released to the host rock.
- Radial cooling occurs after crystal lock-up (CVF < 0.5), when the magma body cools conductively from the outside inward with fluid transfer dominated by capillary fracturing. During this stage, the system forms concentric rings of tube-flow and fracturable mush. Exsolved fluids are assumed to use this dynamically increased permeability to flow to the cupola zone and be released to the host rock. A numerical representation of that process has been described by Ref^19^.

### Governing equations and initial conditions

The coupled magmatic-hydrothermal model simultaneously solves for magma convection within the reservoir (Navier-Stokes equations), fluid flow in the host rock (Darcy’s law), heat conduction and latent heat release^22^.

Magma convection is governed by an implementation of the Navier-Stokes equations with the momentum conservation equation:

1 $$\begin{aligned} \rho _{0} \frac{\partial u}{\partial t}=\mu \nabla ^{2} u-\nabla P-\rho _{0} u \cdot \nabla u-\rho g \end{aligned}$$

and the continuity equation for incompressible magma:

2 $$\begin{aligned} \nabla \cdot u=0 \end{aligned}$$

Conservation of energy within the magma reservoir is obtained by solving:

3 $$\begin{aligned} \frac{\partial T}{\partial t}=u \cdot \nabla T+\nabla \cdot \frac{K}{\rho _{0} c_{p}} \nabla T-\frac{L}{c_{p}} \frac{\partial F}{\partial t} \end{aligned}$$

with temperature *T*, specific heat capacity $c_{p}=880~\textrm{J}~\textrm{kg}^{-1 \circ }\textrm{C}^{-1}$, latent heat of crystallization $L=3.5 \cdot 10^{5}~\textrm{J} ~\textrm{kg}^{-1}$, thermal conductivity $K=2~\textrm{W}~\textrm{m}^{-1 \circ }\textrm{C}^{-1}$, and melt fraction *F*.

In the permeable host rock, Darcy flow of multi-phase compressible saline fluids ($H_{2}O + NaCl$) is calculated as:

4 $$\begin{aligned} v_{i}=-k \frac{k_{r, i}}{\mu _{i}}\left( \nabla p-\rho _{i} g\right) , \quad i=l, v \end{aligned}$$

where *k* is the bulk rock permeability, $k_{r}$ the relative permeability of phase $i, \mu _{i}$ the dynamic viscosity, *p* fluid pressure, and $\rho _{i}$ the density of phase *i*. For the mobile phases, a linear relative permeability model is applied with $k_{r v}+k_{r l}=1-S_{h}$, the saturation of an immobile solid halite phase $S_{h}$, the residual saturation $R_{l}=0.3\left( 1-S_{h}\right)$ for the liquid phase and $R_{v}=0.0$ for the vapor phase.

Conservation of fluid mass, salt mass and energy are calculated as:

5 $$\begin{aligned} & \frac{\partial \left( \phi \left( S_{l} \rho _{l}+S_{v} \rho _{v}+S_{h} \rho _{h}\right) \right) }{\partial t}=-\nabla \left( v \rho _{l}\right) -\nabla \left( v_{v} \rho _{v}\right) +Q_{H_{2} O+N a C l} \end{aligned}$$

6 $$\begin{aligned} & \frac{\partial \left( \phi \left( S_{l} \rho _{l} X_{l}+S_{v} \rho _{l} X_{v}+S_{h} \rho _{h}\right) \right) }{\partial t}=-\nabla \left( v_{l} \rho _{l} X_{l}\right) -\nabla \left( v_{v} \rho _{v} X_{v}\right) +Q_{N a C l} \end{aligned}$$

7 $$\begin{aligned} & \frac{\partial \left( (1-\phi ) \rho _{r} h_{r}+\phi \left( S_{l} \rho _{l} h_{l}+S_{v} \rho _{v} h_{v}+S_{h} \rho _{h} h_{h}\right) \right) }{\partial t}= \nonumber \\ & \quad \nabla (K \nabla T)-\nabla \left( v_{l} \rho _{l} h_{l}\right) -\nabla \left( v_{v} \rho _{v} h_{v}\right) +Q_{e}. \end{aligned}$$

with the porosity $\phi$, the mass fraction of $\textrm{NaCl}$ $X_{i}$, the specific enthalpy $h_{i}$, the source terms $Q_{\textrm{H}_{2} \textrm{O}+\textrm{NaCl}}, Q_{\textrm{NaCl}}$ and $Q_{e}$, and the subscript *r* for the rock.

The numerical approaches for fluid and magma flow have both been benchmarked in previous studies^22,34^. The calculation of dynamic magma, fluid and rock properties follows the descriptions in Ref^21^. The energy conserving equations (Eqs. 3 and 7) are coupled through the respective heat conduction terms in dependence on the thermal conductivity *K*, which controls the heat transfer from the magma reservoir to the host rock, forming a conduction-dominated zone with no magma and fluid flow at temperatures between crystal lock-up at CVF = 0.5 and the brittle-ductile transition, where permeability is gradually increased (Fig. 1d). Due to the non-linear nature of dynamic magma viscosity on the one side and dynamic permeability on the other side, the evolution of this zone is affected by the mesh resolution. Vigorous convection in low-viscosity, low-crystallinity magmas can lead to highly transient, thin thermal plumes that would require very fine mesh resolutions. For the present simulations, we use a mesh resolution that is coarse enough to be computationally feasible and fine enough to reduce mesh effects and capture the main effect of heat transfer by magma flow from the center to the rim of the reservoir at intermediate-crystallinity conditions^22^.

To investigate the interplay of coupled processes controlled by non-linear properties of melts, rocks and fluids, we use a generic set-up including a stylized magma reservoir with a total volume of about 50 $\mathrm {~km}^{3}$, commonly assumed to be the minimum size for porphyry copper systems. Previous simulations resolving incremental sill emplacements show that such reservoirs can be built up with magma injection rates on the order of $10^{-2} \mathrm {~km}^{3} / \textrm{yr}$^13^. For this study, the simulations focus on volatile degassing which starts at intermediate crystallinities of CVF = 0.4. To obtain realistic initial boundary conditions for this stage of magma degassing, the model first calculates an incubation phase, where conductive heat transfer from an initially $970\;^{\circ }\textrm{C}$ hot magma reservoir to the host rock generates a low-permeability conductive boundary layer around the intrusion. During this incubation phase, which lasts about 10-15 kyrs depending on the intrusion depth (Fig. 2), magma convection thermally homogenizes the reservoir at CVF < 0.4. The subsequent degassing phase from a mush state (CVF < 0.4) now starts at conditions with a conductive boundary layer around the reservoir of 1-2 km thickness (see $400\;^{\circ }\textrm{C}$ isotherm Fig. 2). At these conditions of intermediate crystallinities (0.4 < CVF < 0.5), magma viscosity has already increased substantially, leading to less vigorous convection as compared to the incubation phase.

### Fluid release from tube-flow and fracturable mush

We calculate crystallization as $\mathrm {X = 1 - \left( \frac{T - T_{sol}}{T_{liq} - T_{sol}}\right) }^{b}$ with a temperature for liquidus and solidus of $\mathrm {T_{liq}} = 1000\;^{\circ }\textrm{C}$ and $\mathrm {T_{sol}} = 700\;^{\circ }\textrm{C}$, respectively, and b = 0.4. For the degassing model, we further take the respective densities of melt, crystals and volatiles into account^21^, which leads to varying temperatures for the transitions between the different magma stages in dependence on pressure and water contents.

The ternary diagram for magma evolution (Fig. 1b) defines sharp transitions between suspension (blue), tube-flow mush (red), impermeable mush (green) and fracturable mush (black). These transitions lead to strong variations in fluid release rates when large parts of the modelling domain reach the respective thresholds at CVFs of 0.4 and 0.7.

Fluids can first be released from the field of tube-flow mush (red), with volatile volume fractions (VVFs) above the percolation threshold between P1 and P2. The self-organization of magma reservoirs with high water contents into tube-flow outburst events (direct transition from blue to red field in Fig. 1b at CVF = 0.4) provides a challenge for the dynamic permeability model (Fig. 1d). The high fluid injection rates lead to extreme increases in fluid pressure at the cupola region, in particular if 3D fluid focusing effects are considered.

The relative extent into the third dimension of the domain is given by a user-defined scaling parameter, which we set to $f_{3 D}=0.25$ for this study. With this scaling factor, we control the size of the magma and the domain in a three-dimensional geometry within a two-dimensional simulation domain. For a radial factor $f_{3 D}=0.25$, the domain with 30 km width extends 7.5 km in the unresolved 3rd dimension. With this scaling, the $10-\textrm{km}$ wide reservoirs of Fig. 1a have an elongated shape with an extent of 2.5 km in the non-resolved third dimension, resulting in a total volume of ca. 50 $\textrm{km}^{3}$ of the intrusion. Volatile degassing is prescribed to occur across the cupola region with a radius $r=300 \mathrm {~m}$ and an area $A_{\text{ cupola } }=\pi r^{2} f_{3 D}$^16,21^.

The model imposes a maximum permeability of $k_{\max }=10^{-13} \mathrm {~m}^{2}$, which is representative for a disturbed crust at several km depth^35^ and restricts the rates at which magmatic fluids can be released from the magma reservoir. Using the mass balance equation, we can estimate the maximum injection rate $Q_{\max }$ that can maintain a fluid pressure gradient $\nabla p_{\text{ fluid } }$ equal to the lithostatic pressure gradient $\nabla p_{\text{ lithosatic } }=\rho _{\text{ rock } } \cdot g$ as:

8 $$\begin{aligned} Q_{\max }= & v_{\max } \cdot \rho _{\text {fluid}} \cdot A_{\text {cupola}} \end{aligned}$$

9 $$\begin{aligned} v_{\max }= & \frac{k_{\max }}{\mu } \cdot \left( \nabla P - \rho _{\text {fluid}} \cdot g\right) \end{aligned}$$

10 $$\begin{aligned} Q_{\max }= & k_{\max } \frac{\rho _{\text {fluid}}}{\mu _{\text {fluid}}} \cdot \left( \rho _{\text {rock}} - \rho _{\text {fluid}}\right) \cdot g \cdot A_{\text {cupola}} \end{aligned}$$

Fluid density and viscosity are calculated for TPX-conditions of the injected fluid at the cupola^36,37^. Furthermore, the model ensures that volatile release can only start when the cupola region has reached a mush state, even if fluid migration has already started at deeper levels within the reservoir. This approach reflects real-world conditions where magmatic fluids are transported within the domain of tube-flow mush towards the cupola and stored in a bubbly foam layer within the magma chamber. Gradual fluid release acts as a buffer, smoothing outburst durations and extending volatile release.

As a second modification to further mitigate the mesh-dependent fluctuations in outgassing behavior at CVF $=0.7$ observed in previous modelling studies^21^, which are related to the sharp transition from tube-flow (red) to fracturable mush (black), we now gradually lower the percolation threshold for fluid release from fracturable mush during further crystallization from P2 to P3.

### Parameterization of permeability increase during brecciation

After fluid release, the host rock responds with dynamic permeability variations due to hydraulic fracturing according to Fig. 1d (for details see ref^38^). The dynamic permeability model relies on an ad-hoc parameterization of incremental permeability increase at fluid pressures $p_{\text{ fluid } }$ exceeding a calculated failure criterion $p_{\text{ fail } }$^16^. In the original model, permeability increases used a dependence on $\left( \frac{p_{\text{ fluid } }}{p_{\text{ fail } }}\right) ^{2}$. To allow faster permeability generation by hydrothermal brecciation, we modified this parametrization to a log-quadratic dependence with

12 $$\begin{aligned} \log \left( k_{t+d t}\right) =\log \left( k_{t}\right) +f_{k p c}\left( \left( \frac{p_{\text{ fluid } }}{p_{\text{ fail } }}\right) ^{2}-1\right) \end{aligned}$$

This parameterization extends the relationship in Figure 9 of ref^39^ where an increase in pore fluid factor translates into an increase in log permeability to hydraulic fracturing at $p_{\text{ fluid } }>p_{\text{ fail } }$ and can now be modified by the user with a conversion factor for permeability-pore-fluid-coupling $f_{k p c}\left( \textrm{m}^{2}\right)$.

The parmaterization requires that fluid pressures in access of the failure criterion generate permeability and a steep fluid pressure gradient to allow magmatic volatiles to ascend. As a post-processing step, we calculate the amount of additional porosity this excess pressure would generate, which provides a measure for the degree of brecciation^40^.

The dynamic permeability model assumes that the brittle crust is near-critically stressed with hydraulic fracturing occurring at near-hydrostatic fluid pressures. Differential stress is gradually reduced along the brittle-ductile transition, leading to hydraulic fracturing of nominally ductile rock at fluid pressures with near-lithostatic values. In natural systems, magma emplacements may influence the stress field around the reservoir, which could affect fracturing and fluid focusing ore even dike injections and volcanic eruptions. Capturing such mechanisms will require the implementation of additional processes into the coupled model. However, we expect that the first-order processes studied in this contribution will still be valid in more complex systems.

### Fluid-melt partitioning of copper and salt

To study the interconnected behavior of outgassing of magmatic volatiles and chemical enrichment of ore elements during crystallization, we added functionality for fluid-melt partitioning of metals and salt. We use constant distribution coefficients to study the fate of ”enthusiastically fluid-loving” ($\textrm{D}=50$) and ”reluctantly fluid-loving” ($\textrm{D}=5$) chemical components^9^. As a consequence, $\textrm{D}=50$ leads to early copper partitioning into the fluid, while $D=5$ reflects enrichment later in the crystallization process. For simplicity, we assume that Cu and NaCl have the same D values, which also leads to varying salinities of the outgassing fluid and can affect the evolution of the hydrothermal system.

Applying constant values for the distribution coefficient D is a simplification intended to capture the first-order control of fluid-melt partitioning during the evolution from early to late degassing following the schematic example^9^. Distribution coefficients can vary with pressure, temperature and melt composition^9^ and can be particularly redox-sensitive in the case of copper^7^. Capturing the complexity of these thermodynamic interactions is beyond the scope of this study, but could be incorporated into the model with further developments.

Copper precipitation from these released saline, metal-rich magmatic fluids in the hydrothermal system is modelled to occur between 350 and $450 ^{\circ }\textrm{C}$^16^, which is typically inferred for mineralization in porphyry copper deposits.

This temperature window for Cu mineralization also generally agrees with numerical simulations using proxies for temperature- and salinity-dependent solubilities of Cu^17,18^. An accurate description of all relevant chemical fluid-rock reactions will require to incorporate full reactive transport modeling and to develop an internally consistent thermodynamic database for the relevant ranges in temperature, pressure and composition.

## Data Availability

The results presented in this publication and additional descriptions of the data sets and software architecture are available through the GFZ Data Services repository. It includes modelling output files in VTU and CSV formats, which can be used to reproduce the figures. For any request regarding access to the data, please contact the corresponding authors.
